# Risk and prediction of multiple primary malignancies after early gastric cancer

**DOI:** 10.3389/fonc.2023.1205358

**Published:** 2023-07-25

**Authors:** Na Chen, Lei Shi, Jian Ge, Ruzhen Jia, Junmei Jiang

**Affiliations:** Department of Gastroenterology, Shandong Provincial Hospital Affiliated to Shandong First Medical University, Jinan, Shandong, China

**Keywords:** early gastric cancer, multiple primary malignancies, nomogram, predictive factor, predictive tool

## Abstract

**Background:**

Patients with early gastric cancer have increased risk of developing multiple primary malignancies (MPM) due to improved survival rates. The purpose of this study was to evaluate the clinicopathological features of MPM and to generate a useful tool for predicting the development of MPM after early gastric cancer.

**Methods:**

We selected 1025 early gastric cancer patients with complete medical records for a retrospective analysis. The Cox proportional risk regression model was used to analyze the independent risk factors for the development of MPM in early gastric cancer. RStudio software was used to compare the OS of early gastric cancer patients with and without MPM, and a nomogram was established to predict the probability of MPM 1-, 2-, 3-year after early gastric cancer. The predictive effectiveness of the nomogram was evaluated by the C-index and calibration curve. Decision curve analysis (DCA) measured the applicability of the nomogram to clinical practice.

**Results:**

Of the 1025 patients with early gastric cancer, 66 patients (6.4%) had 69 primary cancers other than early gastric cancer. They had a median follow-up of 41 months, and their cumulative incidence of MPM was 4.9%, 5.4% and 5.9% after 1-, 2-, and 3- year, respectively. Oesophageal cancer was the most frequently detected MPM, followed by lung and colorectal cancers. Male (p=0.038), age ≥65 years (p=0.003), smoking history (p=0.036), and lymph node metastasis (p=0.013) were independent risk factors for MPM in patients with early gastric cancer. Patients with early gastric cancer with MPM had a worse OS prognosis than patients with early gastric cancer without MPM (p<0.001). The internally validated nomogram predicted the probability of developing MPM after early gastric cancer (C index= 0.697). The calibration chart showed that the predicted probability of MPM in early gastric cancer was similar to the observed result, and the DCA showed strong clinical practicability.

**Conclusion:**

After the diagnosis and treatment of early gastric cancer, we should be alert to the possibility of MPM and perform regular and careful monitoring.

## 1 Introduction

Gastric cancer is the fifth most common malignant tumour in the world and ranks fourth in global cancer mortality rate after lung cancer, colorectal cancer and liver cancer (1, 2). There are approximately 1.2 million new cases of gastric cancer each year worldwide, of which China accounts for approximately 40%. Early gastric cancer accounts for approximately 20% of cases at diagnosis, and the 5-year survival rate after endoscopic resection can reach 92.6% (3). Multiple primary malignancies (MPM) are two or more primary malignancies occurring simultaneously or consecutively in the same individual, which can originate from the same organ, paired organs, different parts of the same system, or different organs of different systems (4–7), although they can display similar histologic subclassifications, each with different clinical features.

In the last two decades, the detection and survival rates of patients with gastric cancer have increased significantly due to the widespread availability of endoscopic screening programs and the development of minimally invasive endoscopic techniques, and the improved prognosis of gastric cancer combined with the continued influence of genetic and behavioural risk factors has led to an increase in the incidence of associated MPM (8, 9). The incidence of gastric cancer with a second primary malignancy (SPM) is 0.7-11% (10–18), and SPM associated with gastric cancer is usually colorectal, lung and liver cancers (8, 9). Studies on early gastric cancer-associated MPM are scarce and not recent, particularly lacking in minimally invasive endoscopic techniques. The prognosis of early gastric cancer is improving, but clinicians may pay attention to late tumour recurrence and metastasis while ignoring the occurrence of early tumour MPM. When symptomatic MPM is diagnosed, it is usually more advanced than asymptomatic MPM, and the patient’s performance status may not allow for the best cancer treatment, resulting in poor prognosis and short survival time for patients. Therefore, it is important to recognize the clinical and pathological features of patients with MPM associated with early gastric cancer in order to detect MPM early enough for treatment to increase patient survival and improve patient quality of life.

The purpose of this study was to determine the clinicopathological features and outcomes of MPM and to analyse the risk factors for the development of MPM associated with early gastric cancer, with the aim of generating a useful predictive tool for the risk of developing MPM in patients with early gastric cancer. Such a tool would help clinicians identify the clinical course and prognostic factors of early gastric cancer patients with concurrent and metachronous primary cancers, which would facilitate to provide early prevention and diagnosis of MPM.

## 2 Methods

### 2.1 Patient and case data

From 1 January 2010 to 31 March 2022, among 1355 patients with early gastric cancer confirmed by gastroscopy and pathological biopsy at the Provincial Hospital of Shandong First Medical University, we retrospectively analysed the medical records of 1025 patients with clinicopathological features of early gastric cancer who had adequate medical records and were available for postoperative follow-up. Early gastric cancer refers to cancerous tissue limited to the gastric mucosal layer or submucosal layer, regardless of its extent or whether there is lymph node metastasis. Exclusion criteria: (1) endoscopic and pathological confirmation of submucosal tumours, including smooth muscle tumours, mesenchymal tumours, fibrous nerve sheath tumours, neuroendocrine tumours, odoriferous pancreas, lipomas, cysts, capillary haemangiomas, etc.; (2) pathological confirmation of low-grade intraepithelial neoplasia, mid- to late-stage gastric cancer and residual gastric cancer. Following Warren and Gates (7), the diagnostic criteria for MPM were as follows: (1) each tumour had definite malignant histopathological changes; (2) each tumour had an independent pathological type; and (3) the possibility of invasion and metastasis of the second cancer as the first primary cancer was excluded. A second tumour was defined as synchronous MPM if it was found concurrently with the first tumour or within 6 months; if it was found >6 months later, it was defined as metachronous MPM. The diagnosis of each malignancy in patients with MPM is identified by a histopathologist. Haematologic malignancies were excluded from the study, and only solid malignancies were included.

### 2.2 Observed indicators

The observation indices were data on patients’ age, sex, history of smoking and drinking, lesion location, maximum lesion diameter, gross appearance, degree of differentiation, depth of invasion, degree of surrounding mucosal atrophy, degree of intestinal metaplasia, whether the lesion site was multifocal, and whether there was lymph node metastasis at the time of diagnosis of early gastric cancer, and the type of the MPM.

Occasional smokers were considered non-smokers, and social drinkers were considered non-drinkers. The lesions were divided into the upper 1/3 of the stomach (cardia, fundus), the middle 1/3 (body of the stomach) and the lower 1/3 (sinus, including the pylorus). The size of the lesion was determined based on pathological measurements and is expressed as the maximum diameter of the lesion. Superficial lesions (Type 0) were classified as elevated (0-I), flat (0-II) and depressed (0-III) according to the Paris classification update criteria (19). Type 0-II was divided into three subtypes, 0-IIa, 0-IIb and 0-IIc, which had slight elevation, flatness and slight depression, respectively. Lesions with both slight elevation and slight depression were 0-IIa+IIc. According to the degree of histological differentiation, WHO staging, and Lauren staging, highly and moderately differentiated ductal adenocarcinoma and papillary adenocarcinoma are differentiated types, while hypofractionated adenocarcinoma, indolent cell carcinoma and mucinous adenocarcinoma are undifferentiated types, and the presence of both types makes for a mixed type. The deepest cancer tissue located above the mucosal muscle layer was called intramucosal carcinoma, and a 500 μm vertical distance from the deepest lesion through the mucosal muscle layer was used as the threshold value to distinguish superficial (SM1) and deep (≥SM2) submucosal infiltration. The excised specimens were fixed in 10% formalin and then examined histopathologically. Histological mapping was performed after serial sectioning of endoscopic specimens at 2-mm intervals and surgical specimens at 5-mm intervals. Gastrointestinal pathologists assessed the degree of differentiation, gross appearance, maximum lesion diameter, depth of invasion, degree of surrounding mucosal atrophy, degree of intestinal metaplasia, whether the lesion site was multifocal and lymph node metastasis according to the Japanese Classification of Gastric Cancer.

### 2.3 Follow-up

After treatment, patients with early gastric cancer visited the outpatient clinic for follow-up every 3 months in the 1st year and then at intervals of 6-12 months. Follow-up examinations included physical examination, blood analysis, ultrasonography, computed tomography (CT) scan and endoscopy. MPM was evaluated by ultrasonography, computed tomography (CT), positron emission tomography (PET-CT), endoscopy, and histological biopsy. Vital status (all-cause mortality) was assessed by case analysis and telephone follow-up. The follow-up period was defined as the number of years from the diagnosis of early gastric cancer to the date of all-cause death, the date of MPM diagnosis, or the date of the last follow-up visit, whichever occurred first. When we considered the risk of MPM, death was a competing event. Unlike deletion, competing events could not occur at the same time as the event of concern. Overall survival (OS) was defined as the time from diagnosis to death or to the last follow-up visit for patients with early gastric cancer without MPM and the time from MPM diagnosis to death or to the last follow-up visit for patients with early gastric cancer with MPM.

### 2.4 Statistical analysis

In this study, continuous variables are described as means with standard deviations, and categorical variables are expressed as numbers with percentages. Age and sex conformed to a normal distribution and were compared by the one-sample t test, comparisons between categorical variables were made using the chi-square test or Fisher’s exact test, and rank data and non-normally distributed variables were compared using the Wilcoxon signed rank sum test. Risk factors for the development of MPM in patients with early gastric cancer were analysed using Cox regression models, and differences were considered statistically significant when P<0.05. RStudio software was used to see whether there was a statistically significant difference in the OS of early gastric cancer patients with vs. without MPM. Nomogram plots were constructed based on the prediction models established using the results of the Cox multifactorial regression model analysis. The predictive power of the models was evaluated using the consistency index (C-index), and calibration plots were drawn to validate the nomogram. Decision curve analysis (DCA) was done to quantify the applicability of the nomogram to clinical practice. All statistical analyses were analysed using SPSS 26 (IBM Corporation, Armonk, NY, USA) and RStudio (2023.03.0).

## 3 Results

### 3.1 MPM and overall survival

A total of 1025 patients with early gastric cancer were included in this study, 66 (6.4%) of whom had 69 primary cancers other than early gastric cancer (**Table 1**), 47 (68.1%) had metachronous MPM, and 22 (31.9%) had concurrent MPM. There were 24 cases of oesophageal cancer, 18 cases of lung cancer, 14 cases of colorectal cancer, 5 cases of pharyngeal cancer, 2 cases of hepatobiliary cancer, and 2 cases of prostate cancer. The mean time interval between the diagnosis of early gastric cancer and the diagnosis of MPM was 10.06 months. The cumulative incidence of MPM after treatment of early gastric cancer was 4.9% at 1 year, 5.4% at 2 years, and 5.9% at 3 years.

**Table 1:** Site of MPM after diagnosis of early gastric cancer.

Site of MPM	Total n=69	Metachronous n=47	Synchronous n=22
Colorectal cancer	14	10	4
Oesophageal cancer	24	18	6
Hepatobiliary cancer	2	1	1
Prostate cancer	2	0	2
Laryngeal Cancer	5	4	1
Lung cancer	18	11	7
Bladder cancer	1	0	1
Renal cell cancer	2	2	0
Thyroid Cancer	1	1	0

**Figure 1:**
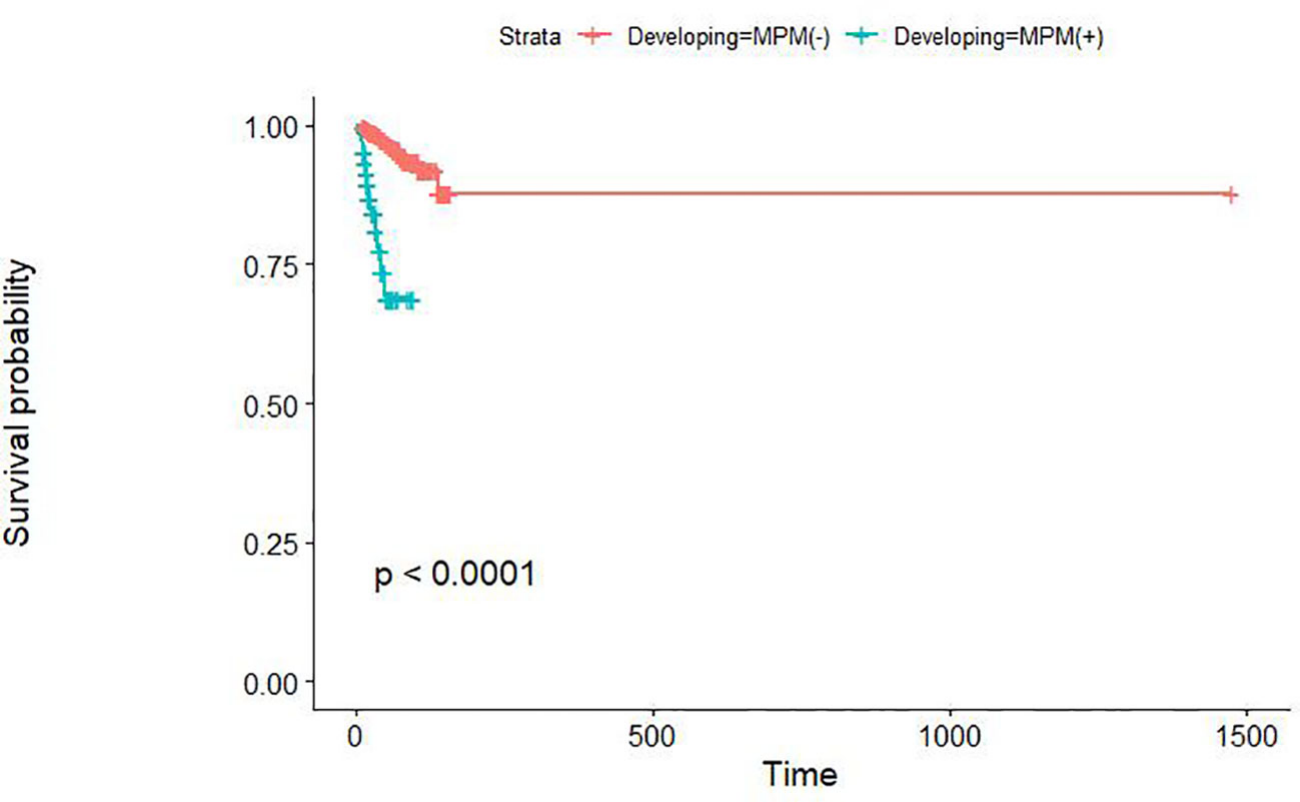
The overall survival of early gastric cancer patients with and without MPM.

Early gastric cancer patients without MPM had a median survival of 43 months and a 3-year OS rate of 98.3%, while the early gastric cancer patients with MPM had values of 23.50 months and 77.7%, respectively. The overall survival rate of patients with MPM was significantly lower than that of early gastric cancer patients without MPM (p<0.01) (**Figure 1**). The cause-of-death patterns of early gastric cancer patients with and without MPM were different: 29 of the 960 early gastric cancer patients without MPM died during follow-up, whose cause of death was mainly other chronic diseases or natural death, while 12 of the 66 early gastric cancer patients with MPM died during follow-up, in whom the cause of death was mainly the progression of MPM.

### 3.2 Predictive factors for MPM

**Table 2** compares the clinicopathologic data between the patients with and without MPM. The MPM group had more male (93.9% vs. 76.1%, p=0.001) and older (65.44 ± 7.946 years vs. 62.00 ± 9.525, p=0.004) patients than the group without MPM. The MPM group more often had a history of smoking (68.2% vs. 45.5%, p=0.000) and lymph node metastasis (13.6% vs. 5.3%, p=0.005). In contrast, patients with early gastric cancer without other primary cancers and patients with early gastric cancer with other primary cancers were similar in terms of history of drinking, lesion location, maximum lesion diameter, degree of differentiation, depth of invasion, degree of surrounding mucosal atrophy and intestinal metaplasia, and whether the lesion site was multifocal.

**Table 2:** Clinicopathological characteristics of early gastric cancer according to the presence of MPM.

Parameters	MPM(-) (n= 959)	MPM(+) (n= 66)	P value
Sex			0.001
Male	730(76.1%)	62(93.9%)	
Female	229(23.9%)	4(6.1%)	
Age (years): Mean (SD)	62.00 ± 9.525	65.44 ± 7.946	0.004
≥65	351(36.6%)	36(54.5%)	
<65	608(63.4%)	30(45.5%)	
Smoking history			0.000
Yes	436(45.5%)	45(68.2%)	
No	523(54.5%)	21(31.8%)	
History of drinking			0.250
Yes	526(54.8%)	41(62.1%)	
No	433(45.2%)	25(37.9%)	
Maximum lesion diameter (cm)	1.561 ± 1.172	1.461 ± 0.815	0.498
Degree of differentiation			0.514
Differentiated	862(89.9%)	61(92.4%)	
Undifferentiated	43(4.5%)	1(1.5%)	
Mixed type	54(5.6%)	4(6.1%)	
Depth of invasion			0.699
M~SM1	816(85.1%)	55(83.3%)	
≥SM2	143(14.9%)	11(16.7%)	
Location			0.098
Upper 1/3	242(25.2%)	21(31.8%)	
Middle 1/3	140(14.6%)	14(21.2%)	
Lower 1/3	577(60.2%)	31(47.0%)	
Gastric atrophy			0.565
Mild or moderate	141(14.7%)	8(12.1%)	
Severe	818(85.3%)	58(87.9%)	
Gross appearance			0.479
0-I	114(11.9%)	10(13.2%)	
0-IIa	166(17.3%)	10(13.2%)	
0-IIb	246(25.7%)	22(28.9%)	
0-IIc	167(17.4%)	11(14.5%)	
0-IIa+IIc	234(24.4%)	18(23.7%)	
0-III	32(3.3%)	5(6.6%)	
Intestinal metaplasia			0.780
Mild or moderate	277(28.9%)	18(27.3%)	
Severe	682(71.1%)	48(72.7%)	
Multiplicity			0.075
No	807(84.2%)	50(75.8%)	
Yes	152(15.8%)	16(24.2%)	
Node metastasis			0.005
Negative	908(94.7%)	57(86.4%)	
Positive	51(5.3%)	9(13.6%)	

M: Intramucosal; SM1: depth of invasion <500 μm from the muscularis mucosae; SM2: depth of invasion ≥500 μm from the muscularis mucosae.

Cox univariate regression analysis was performed on all variables, and variables with p<0.05 were then subjected to Cox multivariate regression analysis, which showed that male sex, age older than 65 years at the time of GC diagnosis, history of smoking and lymph node metastasis were independent predictors of MPM among early gastric cancer patients (**Table 3**).

**Table 3:** Cox univariate and multifactor regression analyses of MPM risk factors.

Parameter	Univariate analysis		Multivariate analysis	
	HR (95% CI)	*P*	HR (95% CI)	*P*
Sex (male vs. female)	4.612(1.678-12.679)	0.003	3.116(1.067-9.099)	0.038
Age (≥65vs.<65) Smoking history	0.481(0.296-0.781) 2.485(1.480-4.172)	0.003 0.001	2.111(1.299-3.430) 1.800(1.038-3.122)	0.003 0.036
History of drinking Maximum lesion diameter (cm) Degree of differentiation Depth of invasion Location Gross appearance Mucosal atrophy	1.346(0.818-2.214) 0.932(0.740-1.174) 0.979(0.356-2.694) 0.835(0.437-1.596) 1.598(0.918-2.781) 0.650(0.203-2.075) 1.350(0.644-2.830)	0.242 0.548 0.494 0.585 0.087 0.527 0.427		
Intestinal metaplasia Multiplicity	1.075(0.625-1.848) 1.627(0.927-2.858)	0.793 0.090		
Node metastasis	2.706(1.339-5.469)	0.006	2.438(1.204-4.939)	0.013

### 3.3 Nomogram

**Figure 2:**
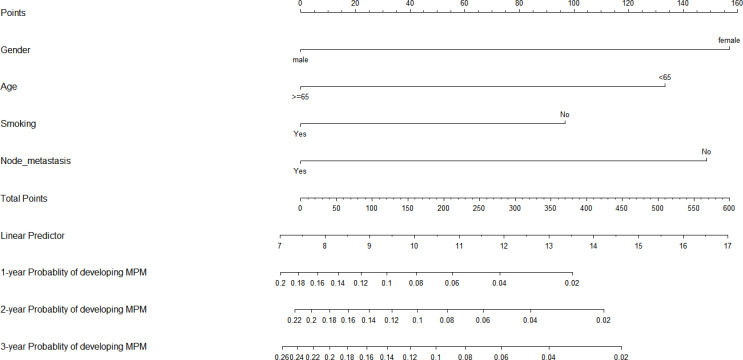
Nomogram for predicting the 1-, 2-, and 3-year probability of developing metachronous MPM.

**Figure 3:**
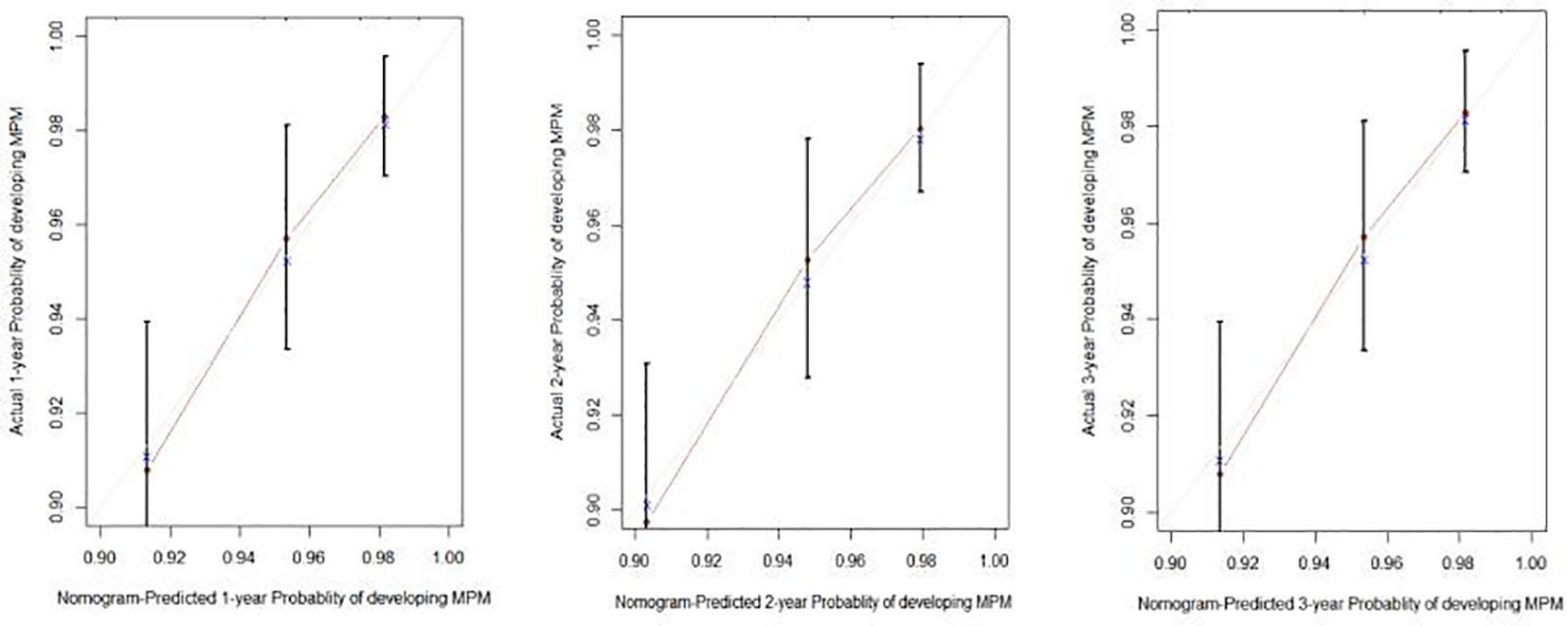
Calibration plots for the 1-, 2-, and 3-year nomogram predictions.

**Figure 4:**
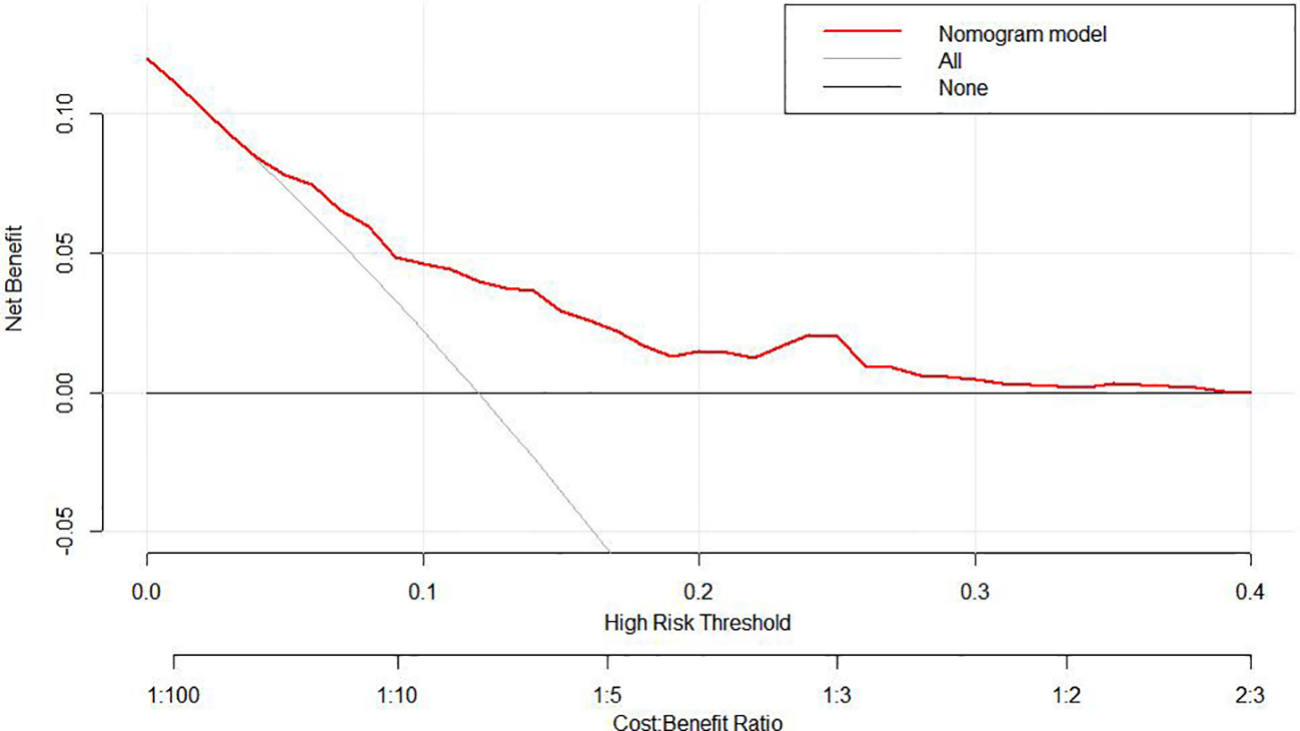
DCA curves of the nomogram for predicting MPM.

To predict the development of MPM, we generated a nomogram (**Figure 2**) based on the results of the Cox regression. From the Cox regression model, male sex (p = 0.038), age over 65 years (p = 0.003), history of smoking (p = 0.036) and lymph node metastasis (p = 0.013) were associated with the development of metachronous MPC. Each variable category in the nomogram corresponds to its respective score evaluation. All the category scores are summed to obtain the total score. By drawing a line vertically downwards from the total score scale point to the horizontal axis, the intersection point is the predicted probability of developing MPM in early gastric cancer patients at 1 year, 2 years, and 3 years. The consistency index of the model was 0.697, and the calibration curves of the nomogram were all similar to or basically fit the 45° diagonal line (**Figure 3**). In addition, the DCA showed strong clinical practicability (**Figure 4**).

## 4 Discussion

With economic and social progress, people have gradually become more aware of matters of health, and advances in active medical examination behaviours and minimally invasive endoscopic techniques have led to a higher detection rate and cure rate of early gastric cancer, with good prognosis. In general, the survival rate has improved, the risk period has grown longer, and the prevalence of MPM in early gastric cancer patients has increased. Domestic and international data indicate that the risk for the development of SPM in the cancer population is twice that of the general population (20, 21). The incidence of multiple primary malignancies associated with early gastric cancer in this study was 6.4%, similar to the results of previous studies (10–18). However, the most common MPM site for gastric cancer in previous studies was colorectal cancer, followed by lung and liver cancer (16, 18, 22–24), while oesophageal cancer was the most common MPM associated with early gastric cancer in the present study, followed by lung and colorectal cancer. In addition to the possible similarity of external risk factors (environment, smoking and drinking history, and diet) (20, 25, 26), we speculate that the predilection for these MPM sites may be because the respiratory tract epithelium and digestive tract epithelium, as well as various digestive glands that are specialized forms of digestive tract epithelium, all have endodermal development and are more likely to be influenced by the same genes and environmental signals during embryonic organ development (27). This conjecture needs to be tested by stem-cell-to-organoid modelling.

Our study showed that there was a significant difference in OS between patients with early gastric cancer with MPM and those without MPM. However, the prognosis of MPM patients itself is worse than that of patients without MPM, which may be due not only to their higher tumour load and comorbid conditions but also to higher psychological burden (28, 29). Therefore, most MPM patients still have a poor prognosis even under regular follow-up. Our nomogram suggests that patients at high risk may need more frequent follow-up or more accurate means of follow-up, which would help to detect MPM at an early stage, thus helping patients achieve good treatment results at an early stage and avoiding late detection of MPM, which would lead them to miss the best time for treatment, worsening their prognosis and the quality and duration of survival. The optimal surveillance interval for MPM should be determined in future studies.

Patients with early gastric cancer, especially males, those aged ≥65 years, and those with lymphovascular involvement, tend to have a greater risk of second primary cancers and of recurrence (23, 30–33). Smoking is an independent risk factor for second primary cancer and increases the incidence of second primary cancer in patients with gastric cancer (34–36), in line with our results. Although our nomogram may be biased due to not enough MPM events, this nomogram can provide a more personalized prediction of MPM for early gastric cancer patients than the independent risk factor model. External validation in independent patient groups is needed to improve the accuracy of the nomogram.

There are several limitations of this study. Since it was retrospective, patient information was obtained mainly through case records and telephone follow-up, so recall bias may have occurred because lifestyle changes may come about as the cancer progresses and symptoms appear. We included only the history of smoking and drinking at the time of detection of early gastric cancer and ignored changes in habits after treatment began, but cancer patients may adopt a healthier lifestyle after diagnosis or treatment (37, 38). We also only analysed the presence or absence of a history of smoking and did not analyse individual smoking status. Since this study was a single-centre study, its results cannot be easily extrapolated or generalized. We also did not gather any information on *H. pylori* infection or eradication. The association between *H. pylori* and gastric cancer has been confirmed in several studies, *H. pylori* infection being associated with gastric cancer incidence worldwide (39–45), and one study showed that *H. pylori* eradication significantly reduced the incidence of gastric cancer (by 46%) and reduced gastric cancer mortality (by 39%) (46). Therefore, once *H. pylori* is detected, early eradication therapy is recommended to maximize the prevention of gastric cancer.

In conclusion, the risk of MPM after early gastric cancer treatment is still high. Age ≥65 years, male sex, smoking and the presence of lymph node metastasis are risk factors for MPM in early gastric cancer patients. Since oesophageal and lung cancers are common MPMs and patients with early gastric cancer with MPM have a poor OS prognosis, it is recommended that after the early gastric cancer is cured, in addition to regular monitoring for gastric cancer recurrence, follow-up oesophageal and chest examinations should also be given particular attention. We generated and validated a nomogram to predict the probability of MPM in patients with early gastric cancer. If this tool proves useful to screen for MPM, we can improve the clinical prognosis of patients with early gastric cancer.

## Data availability statement

The raw data supporting the conclusions of this article will be made available by the authors, without undue reservation.

## Ethics statement

Written informed consent was not obtained from the individual(s) for the publication of any potentially identifiable images or data included in this article.

## Author contributions

NC contributed to the study design and wrote the article. LS and JG did the data analysis. NC and RJ generated and improved the figures and tables. LS proofread the manuscript. JJ reviewed the article. All authors contributed to the article and approved the submitted version.
