# Corrigendum: Risk and prediction of multiple primary malignancies after early gastric cancer

**DOI:** 10.3389/fonc.2023.1298412

**Published:** 2023-11-28

**Authors:** Na Chen, Lei Shi, Jian Ge, Ruzhen Jia, Junmei Jiang

**Affiliations:** Department of Gastroenterology, Shandong Provincial Hospital Affiliated to Shandong First Medical University, Jinan, Shandong, China

**Keywords:** early gastric cancer, multiple primary malignancies, nomogram, predictive factor, predictive tool

In the original article **Figure 2** was uploaded incorrectly due to a personal mistake. The corrected **Figure 2** is shown below.

**Figure 2:**
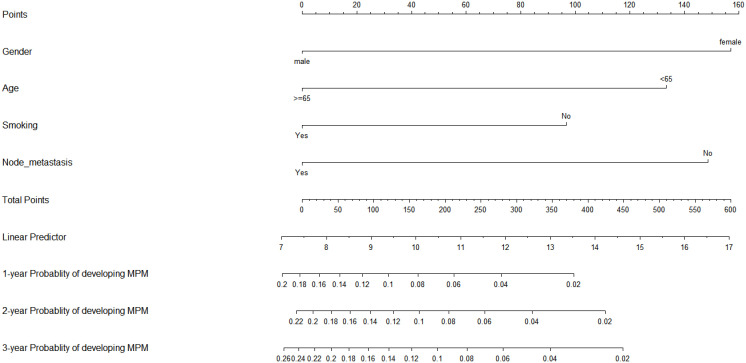
Nomogram for predicting the 1-, 2-, and 3-year probability of developing MPM.

The authors apologize for this error and state that it does not in any way change the scientific conclusions of the article. The original article has been updated.

